# Adolescents’ perception of dietary behaviour in a public school in Chile: a focus groups study

**DOI:** 10.1186/s12889-020-08908-x

**Published:** 2020-05-29

**Authors:** F. Vio, M. Olaya, M. Yañez, E. Montenegro

**Affiliations:** 1 Institute of Nutrition and Food Technology (INTA), University of Chile, El Líbano 5524, Macul, 7830489 Santiago, Chile; 2 Municipal Health Service Los Andes county, Los Andes, Chile

**Keywords:** Adolescents, Community health promotion, Dietary behavior, Qualitative methods, Focus groups

## Abstract

**Background:**

The objective of this study was to assess dietary behavior among sixth- to eighth-grade students to inform the delivery and content of nutrition education.

**Methods:**

This was a qualitative study through focus groups. Subjects were 57 adolescents 10–14 years old, 30 males and 27 females distributed in six groups. To compare group responses, transcriptions were coded using the original question guide. The information was analyzed using the content analysis technique.

**Results:**

The main findings showed that adolescents knew dietary guidelines, but they consumed non-healthy food. They liked to cook but preferred fast food preparations. They increased fast food consumption on weekends and with friends. In utilization of Information Communication Technologies (ICT), all students had access to technology through mobile phones, tablets and computers and were open to have an interactive program with personal information about diet and behavior.

**Conclusions:**

Adolescents dietary behavior is not healthy and can be changed with interactive programs considering participation, personal information and utilizing ICT.

## Introduction

Childhood overweight and obesity is increasing in Chile [1] and worldwide [2]. This increase includes school-age adolescents from sixth to eighth grade (age 10–14). The 2015 National Study of Physical Education [3] showed that 55% of eighth-grade students in Chile had a normal BMI, 25% were overweight and 20% obese. The National Board for School Assistance and Scholarships (Junta Nacional de Auxilio Escolar y Becas-JUNAEB) in their 2016 nutritional map showed that 31.8% of high school freshman were overweight and 13.4% obese, noting that obesity had significantly increased since 2011 when it was 8.2% [4]. This increase depends on several conditions, but the two main factors are inadequate nutrition and sedentary lifestyle. Both issues have been the target of significant interventions in pre-school and primary school until fourth grade since the 1990s in Chile, but these interventions have been isolated efforts, without continuity and have not reduced the problem [5]. In general, adolescents know what they should be eating, but this knowledge has not translated into behavioral improvements in diet [6]. Thus, in order to generate policy proposals, it is necessary to know about adolescents’ current dietary behavior and identify methodologies that drive changes toward healthy eating. The best way to address this issue is throughout focus groups, which provides an in depth knowledge about this phenomenon [7].

Adolescence is a critical point for health, as adolescent face challenges related to choices for their own physical and social environment and begin to make their own eating decisions. It is also a unique opportunity to influence the adoption of healthy eating habits and other positive lifestyles, such as physical activity [8]. Additionally, the use of technology is often useful for healthy eating and disease prevention interventions in this age group. Previous studies have shown that the use of Information and Communication Technology (ICT) in promoting healthy habits has been successful and easy to apply among adolescents, considering their familiarity with electronic devices [9–11].

Educating adolescents is a tough, exciting and challenging task. If done well, it can have extraordinary impact because habits adopted in adolescence may continue across the life course [12]. However, it is noteworthy that while there are many interventions for this age group aimed at reducing the consumption of tobacco and alcohol, there are few in-depth studies in Chilean adolescents related to eating habits and their consequences for obesity [13]. The objective of this study was to assess dietary behavior among sixth- to eighth-grade students through focus groups to inform the delivery and content of nutrition education.

## Methods

### Study design

Qualitative focus groups were conducted to identify eating habits and behaviors, culinary skills and barriers to healthy eating among adolescents. The purpose of a focus group is the controlled production of a group’s discourse among subjects that come together for a limited time, in order to discuss a particular topic proposed by the researchers [14–17].

### Focus groups recruitment

Participants were sixth to eighth-grade students (10–14 years old) of medium-low socioeconomic status measured by the JUNAEB School Vulnerability Index [18] from an urban public school in Los Andes, considering that most of the students are medium-low SES in Chile. The school was selected among the public schools in Los Andes without any relationship between the school/pupils and the researchers.

There were no exclusion criterion and the sample was selected in a non-probabilistic manner among those students who were willing to participate. The sample included 57 adolescents (27 females and 30 males) distributed in six focus groups. The composition of each group was established based on gender and age: 10 sixth-grade males in group 1; 7 sixth-grade females in group 2; 10 seventh-grade males in group 3; 10 seventh-grade females in group 4; 10 eighth-grade males in group 5, and 10 eighth-grade females group 6. Focus groups were conducted out of the class room and teachers did not participate in the discussions.

### Data collection

The focus groups discussions were moderated by a psychologist and an assistant moderator (nutritionist). Each group followed a guided open-ended questionnaire designed and validated in previous studies with parents [6] and teachers [19] using theoretical models adapted for adolescents (Table 1). Items included conceptualizing diet, food intake, eating habits, cooking skills, barriers to a healthy diet and use of ICT. The moderator encouraged all participants to answer each one of the questions. The assistant moderator was present to take notes and facilitate the direction and flow of the discussion. Field notes were used to record contextual details and non-verbal expressions to contribute to data analysis and interpretation. The central theme was broken down into topics that allowed to focus the discussion on a specific dimension according to experience [14]. A self-evaluation was utilized in the item conceptualizing diet with a scale ranged from 1 to 7 (with 7 being the highest) to the own eating habit.

**Table 1:** Focus group guide

Conceptualizing diet	What is a healthy diet? Are your eating habits healthy? On a scale of 1 to 7 (with 7 being the highest), what score would you give your diet? What foods do you consider to be healthy? Unhealthy? Why? Do you know what the dietary guidelines are? What are they?
Food intake	Tells us about your preferences and frequency of eating the following foods: dairy, fruits, vegetables, fish or seafood, bread, sugar-sweetened beverages (juice, soda, others). How many glasses of water do you drink per day (in number)? How many times per week? How many meals do you eat at school (in number)?
Cooking	Do you like to cook? Do you know how to cook? Do you help prepare the meals in your house?
Eating habits	Do you eat breakfast every day? What do you eat? Do you eat lunch every day? At a set time? Is your lunch healthy? Do you eat at tea time? Do you eat dinner every day? What do you eat? Do you eat with other members of your family? Does your diet change on the weekend? Why? Why not? How do your friendships influence your diet?
Barriers to healthy eating	What are the primary difficulties for having a healthy diet? Do you have money to buy foods? Which type of foods? Do you think that advertisement influences what foods you buy? What drives your decision making when choosing what foods to eat? What is the main barrier to eating a healthy breakfast?
Technology	What type of technology do you most use? What social networks do you use most? How could technology be used to teach about eating and nutrition at school?

Each focus group discussion was audio recorded and lasted 50–60 min. The researchers ensured the confidentiality established in the signed consent and explained in detail the purpose of the study to all participants.

### Analysis of focus groups transcripts

After the transcription of focus groups, data was checked for accuracy by the moderator and the assistant moderator reading and re-reading the transcripts in order to achieve an in-depth understanding of the data by the two researchers. The text was reviewed by the responsible investigator for interpreting written documents. It represents an analytical procedure aimed at giving meaning to what is being studied. It is an interpretation in which each datum may have a manifest symbolic meaning, such as conscious feelings, ideas and impulses that contain repressed materials, and/or latent one, existing in unconscious or dormant but potentially able to achieve expression. Moreover, each datum may be emitted by the interviewee consciously or unconsciously. The content analysis scheme was followed to process the data [20]. Some questions were quantified, such as the diet score, the frequency of diet, amount of water consumed, number of meals at school and other were just yes or no. In the case of self-evaluation, each participant was asked to assign a grade from 1 to 7 (with 7 being the highest).

The final step of analysis included interpretation of the categories obtained following the criteria which included level of depth, grouping of answers and discourse establishment by segment. This categorization was utilized by the same authors in another study with teachers utilizing “Majority” and “Minority” groups [19]. This methodology searches for meanings that are regrouped into categories to reflect the most important ideas. In this case we utilize three groups: Majority, Intermediate and Minority groups, depending on the predominant ideas and the percentage of participants.

- Majority group (predominant group with 60–70% of participants): categories appear clearly within individual experiences and can be combined into a clear discourse.
- Intermediate group (with 20–30% of participants): ideas represent a reality that differs sharply compared to the other two groups.
- Minority group (with less than 10% of participants): ideas appear with less force but represent important realities. From a sociological perspective, this group breaks the social consensus that emerges from the other profiles, with proposals against majority rule. The ideas of this group do not always influence other ideas, but rather they generate new approaches.

## Results

### Conceptualizing diet

When adolescents were asked about healthy eating, the answers were clear. Adolescents shared knowledge they had acquired over the years. Responses alluded to concepts such as health, healthy foods, physical activity and water consumption. When asked about their knowledge of healthy versus unhealthy foods, they were easily assigned into one of the three groups.

The Majority group had a minor interest in healthy eating; however, they preferred the consumption of junk food, but considered making changes to their habits.

The Intermediate group were those who cared about their diet. This group reported the importance of consumption of healthier foods than profile 1. Female adolescents stated it was important to “eat in moderation” (eighth grade female) and they were “careful not to overeat” (eighth grade female). On the other hand, males mentioned sports as a primary factor for a healthy life. An example was the opinion of one student: “Exercising ... makes you not want to eat junk ... you just can’t” (seventh grade male).

... I do a lot of exercise ... in my house we don’t eat a lot of fried food and if we eat sweets it’s like once a month ... "(eighth grade female).

Among the Minority group, unhealthy foods were preferred. They did not demonstrate any interest in making a change and were indifferent about personal eating habits (Table 2).

¨ ... I eat healthy when I’m with my grandma and my mom … when I’m with my brother we only eat junk food ... "(seventh grade female)

“... Most of the time I eat junk food. I hardly ever eat healthy [food]...” (Seventh grade male)

The self-evaluation adolescent diet did not differ by sex. However, females were more severe with themselves with an average of 4.4, on a scale of 1 to 7, while males had an average of 5.0.

"... a 3 because sometimes when I'm with my mom I eat fruit with yogurt or milk with banana, but when I'm with my dad I eat junk food ... ¨ (sixth grade female).

“... about a 5 because I eat a lot of bread ... and I do not eat a lot of vegetables” (seventh grade male).

**Table 2:** Conceptualizing diet

	Majority group	Intermediate group	Minority group
Male and Female Adolescents	Moderate intake of fruits and vegetables. High intake of high-calorie foods.	High intake of fruits and vegetables. Concern for healthy eating. Low intake of high-calorie foods.	Minimal intake of fruits and vegetables. Indifference about personal eating habits. High intake of high-calorie foods.

### Food intake

Adolescent diet reflected behaviors far from those recommended in the Chilean population dietary guidelines [21]. For foods such as dairy, fruits, vegetables and fish, frequency of consumption was lower than the daily recommendation, with an average dairy consumption between 1 and 2 servings, eaten primarily at breakfast or snack, 3 servings of fruit and vegetables a day; and 1 serving of fish per week. On the other hand, adolescents exceeded recommended consumption of sugary drinks and bread, consuming 0.5 to 1.0 l of sugar-sweetened beverages and 2 to 3 units of bread. Participant comments focused on the feeling of satiety and taste for these foods. The majority of participants consumed sweet or salty snacks every day. Water consumption reported to be approximately a half liter per day.

“There is always soda in my house. I can drink up to 3 liters ... they buy diet [soda] but I don’t like it, it grosses me out” (eighth grade female).

### Culinary skills

Adolescents report that they like to cook, however they reported cooking foods with quick preparations, like rice, pasta, sandwiches, burgers and fries, among others, and showed little interest and ability to prepare more complex foods.

“When I'm hungry I cook pasta, fries, rice or sausage” (seventh grade female)

“The only thing I know cooking is pasta, egg or hamburger” (Eighth grade female)

### Eating habits

Participants explained their eating habits, considering their daily meal times offered at school (Table 3).

**Table 3:** Eating habits

	Majority group	Intermediate group	Minority group
Breakfast	Eats breakfast daily, at home or at home and school (double breakfast). Frequently eaten foods include: bread with butter, ham, cheese or jam; milk, tea or yogurt with cereal.	Eats breakfast at school. Eats: bread with butter, egg, jam; milk or yogurt with cereal.	Does not eat breakfast. Reports not being hungry in the morning.
Lunch	Eats lunch daily in school. If still hungry after school, eat again at home.	Eats at school.	Eats lunch brought from home or they eat at home.
Tea time/ dinner	Tea time is the main meal with a high bread consumption.	No differences with the other two profiles	Tea time and dinner are a single meal.

In Majority and Intermediate groups most of the adolescents reported eating breakfast at home. However, they felt that their breakfast choices were unhealthy, as they sought the fastest alternative, which was usually a hot drink (tea or milk) and bread. The most frequent healthy breakfast barrier was time, with participants stating that they were unable to prepare something more elaborate.

Both groups ate lunch at school, but some in the Majority group ate again at home after school. They reported not liking the school lunch because it was low in salt. Thus, most participants had the habit of bringing their own salt to improve the taste.

In the Minority group they did not eat breakfast and lunch at school.

“I do not eat breakfast because I get up very late and I do not like to eat early” (sixth grade female).

With regard to tea time (called “once” in Chile) the three groups were similar. Adolescents reported that this was the time when the family ate together with bread being an important part of this meal.

“*At tea time, I eat bread with egg or butter*” (seventh grade female).

On weekends, the majority of adolescents explained that their eating habits changed for the worse, in comparison to during the week, as result of increased junk food consumption. Adolescents stated that on the weekend they go out to eat with their families to places like the mall food court, where they mostly ate burgers, pizzas, and grilled chicken with fries, hot dogs, Chinese food and other “junk” food. On the other hand, a group reported eating healthy and unhealthy foods. The minority group stated that their diet improved on the weekend, because there was more time to cook improving access to “homemade” foods with an increased consumption of vegetables in salads.

Considering the stage of development and importance of the peer group, adolescents were consulted about the foods they normally ate when they attended meetings with their friends. They answered that their food was not healthy, without any difference by sex with a preference for pizza, soft drinks, hot dogs, hamburgers and snacks, among others. When asked if their friends influenced their food choices, the answer of the majority of the group was that their peers influenced their choices. However, some of them recognized that the final decision was their own responsibility.

### Barriers

The taste of high-calorie food (they considered “junk” food to taste good) and the sensation of satiety were mentioned as barriers to healthy eating.

“Because they are better” (eighth grade male)

“Because they fill you up more” (eighth grade male)

They also considered that unhealthy foods are a “vice” and when they start eating they cannot stop (addictive factor). They reported being aware of this situation but were not willing to change their behavior. They considered that these factors were the biggest barriers to eating healthy.

“Once you eat you cannot stop” (male, seventh grade)

“It’s that yummy things are not healthy” (female, eighth grade)

Another barrier was the low-salt lunch offered by the school cafeteria.

*“The lunch that they have here doesn’t have enough salt, for that reason lots of us bring our own” (female, sixth grade)*

### Use of Technology

All students had access to technology through mobile phones, tablets or computers. The most used social networks were (in order) Facebook, Whatsapp, Youtube and Instagram. The technological device most used by students was a cellular phone with internet access. Adolescents were asked if they would use technology for food education with some examples, such as messages with dish recipes and other. Answers varied, with students noting that their interest in the use of technology for food education would depend on the content, such as a personalized program with information like weight and height, for a self-assessment of nutritional status or the benefits of engaging in various sports (Table 4). However, in a previous study with teachers, they pointed that the utilization of ICT should be limited, so that the child cannot open programs on the tablet or computer, as occur in computer classes where the children use other programs while they are in classes [19].

**Table 4:** Adolescents’ thoughts on a technology-based nutrition intervention

Females	Males
Interactive program with educational games. Each adolescent must have their own tablet or computer. A variety of games should be offered Healthy but good-tasting recipes. Should include music and tutorials. Information on healthy diets. Dance choreography.	Interactive program. Personalized program that incorporates personal information, like weight and height for a self-assessment of nutritional status, or the benefits of engaging in various sports. Attractive colors and designs. Personalized alarm that indicates when to eat.

## Discussion

The need to confront the increase of overweight and obesity in Chilean adolescents is clear. In Chile, some nutritional programs have been implemented from a health promotion point of view in preschool and primary school-age children. However, adolescence has not been a period typically targeted by these interventions [5].

Education on eating habits designed for teenagers could contribute to changes in eating behavior that prevail over the life course [22]. However, before any intervention begins it is important to identify behaviors and habits among sixth to eighth graders, as has been done previously among younger students [6].

In this study, we found that adolescents had information on healthy and unhealthy eating and easily recognized the foods that correspond with each category. However, changing information into habits is difficult. These findings are consistent with studies from the US and Europe in which associations between nutritional knowledge and behavior was not found among adolescents who attribute their weight status to non-modifiable factors [23, 24].

The barriers to healthy eating highlighted by adolescents were consistent, with time playing an important role, both in the foods they chose to eat and foods they prepared. Similar to our study, others have identified that time, usefulness and family preferences are all barriers to healthy eating [25]. To cope with this problem, adolescents’ interventions could be participation-based, with practical activities such as cooking workshops, school gardens with vegetables, recycling and other practical activities which adolescents can replicate at home.

In general, healthy food consumption is not a frequent behavior among adolescents. The result of the 2013 Global School-based Student Health Survey (GSHS) in 13–15 years old students showed that just 42.5% of the students ate fruit twice a day and 24.9% ate vegetables three times per day in the last 30 days [26].

As in other studies, our results show the importance of peers in food choices, which can be used to generate affinity and acceptance in a social group [27]. This was an important finding in the focus groups: when they were hanging out with friends in the food garden at the malls a great deal of unhealthy food was consumed, resulting in a high intake of calories, fats and carbohydrates. It is important to point out that the majority of adolescents reported eating breakfast and lunch twice, at home and at school. These results are similar to those reported in a recent study showing that 20% of schoolchildren ate breakfast twice and 33% ate a double lunch [28].

Finally, we found that technology was widely used, primarily via cellphone. Facebook, Whatsapp, and YouTube were the most frequently used applications. Adolescents reported that they might be interested in an application designed to teach about healthy eating, with some differences by sex. Females preferred healthy recipes that would be easy to prepare and liked the idea of activities like dancing or those aimed at weight management. Males preferred sports and personalized information on weight and height. Both males and females indicated that it would be important to have an interactive program that used personal information. In this way, technology would offer an opportunity for improving adolescents’ eating habits [29].

### Strengths and limitations

The main strength of this study was identifying adolescent behavior using an in-depth methodology, which was well accepted by the participants. Questions in the semi-structured topic guide were based in previous studies and allowed for coherent conclusions. However, qualitative data has limitations and more studies with quantitative and qualitative data should be conducted. Among the limitations there exists the difficult to the generalizability of the results, taking into account that this study was conducted in a Chilean city with a specific methodology which includes the background and reflexivity of the researchers, which can introduce potential bias in the conduction of the study and/or in the analysis of data.

## Conclusions

Adolescents know what constitute eating healthy habits, but do not practice these habits. A healthy diet is important for daily life and for future health and nutrition. Based on evidence of effectiveness of targeted interventions to change eating habits, there is a need to design an education program aimed at using technology to support dietary changes in adolescents. Considering and understanding adolescents’ current habits, preferences and behaviors is the only way to create strategies for the prevention of overweight and obesity, and to propose public policies for this age group.

## Data Availability

The datasets used and/or analyzed during the current study are available from the corresponding author upon request.

## Abbreviations

JUNAEB: Junta Nacional de Auxilio Escolar y Becas (The National Board for School Assistance and Scholarships).
ICT: Information and Communication Technology
